# Unlocking the Secrets of Andersen–Tawil Syndrome: The Role of Next-Generation Sequencing in a Family With Long QT Syndrome

**DOI:** 10.1155/crp/9532818

**Published:** 2025-11-10

**Authors:** Mansoor Namazi, Niloofar Naderi, Amir Askarinejad, Mohammad Dalili, Majid Maleki, Samira Kalayinia

**Affiliations:** ^1^Cardiovascular Research Center, Rajaie Cardiovascular Institute, Tehran, Iran; ^2^Cardiogenetic Research Center, Rajaie Cardiovascular Institute, Tehran, Iran

**Keywords:** Andersen–Tawil syndrome, arrhythmia, *KCNJ2*, likely pathogenic, long QT syndrome, variant

## Abstract

**Background:**

Andersen–Tawil syndrome (ATS) is a rare inheritable potassium channelopathy, accompanied by ventricular arrhythmias due to long QT intervals, muscle weakness, and dysmorphic features. Next-generation sequencing can identify the genetic causes of the phenotype.

**Methods:**

Whole-exome sequencing (WES) was performed on a 12-year-old girl with long QT syndrome and dysmorphic features. Sanger sequencing was subsequently used to confirm the variant and perform segregation analysis in the proband and all available family members.

**Results:**

WES identified a novel homozygous likely pathogenic missense variant (chr17, c.G598A, p.V200M; hg19; NM_017755.5) in *KCNJ2* in the proband. Some of her family members were heterozygous for the variant but remained asymptomatic with no cardiac manifestation.

**Conclusions:**

We propose that patients with dysmorphic skeletal findings and cardiac arrhythmias be evaluated via NGS for possible genetic variants.

## 1. Introduction

Andersen–Tawil syndrome (ATS) is a multisystem disorder with an autosomal dominant/recessive pattern [1]. The prevalence of ATS is about 1/1000000, and the concomitant involvement of cardiac rhythms (frequent ventricular ectopic activities, sustained ventricular arrhythmias, and prolonged QT intervals), muscles (periodic paralysis and weakness), and skeletal bones (prominent craniofacial dysmorphic features) is known as the ATS triad [2, 3]. Variants in *KCNJ2* and *KCNJ5*, expressed in the heart, bones, and muscles, constitute the principal genetic cause of ATS presentation [1]. Patients with these variants may exhibit different symptoms that could be part of the triad or may remain asymptomatic. The variant phenotypes of *KCNJ2* and *KCNJ5* are called “ATS Type 1” and “ATS Type 2” [4]. *KCNJ2* encodes the α-subunit of the inwardly rectifying potassium (K+) channel Kir2.1 and participates in the inwardly rectifying K+ current (IK1), classified as long QT syndrome Type 7 [5]. About 60%–70% of patients with ATS exhibiting channelopathies have the *KCNJ2* variant, which encodes the *α* subunit of the K+ channel protein, Kir2.1, resulting in complete loss of function in relative K+ channel functions [6]. In addition, approximately 30% of ATS cases are *de novo* and sporadic, indicating that unidentified genes are a factor in their presentation [7].

Here, for the first time, via next-generation sequencing (NGS), we identified a novel likely pathogenic homozygous variant in the *KCNJ2* gene, c.G598A: p.V200M, in an Iranian girl with ATS.

## 2. Methods

### 2.1. Family Recruitment and Clinical Characteristics

**Figure 1:**
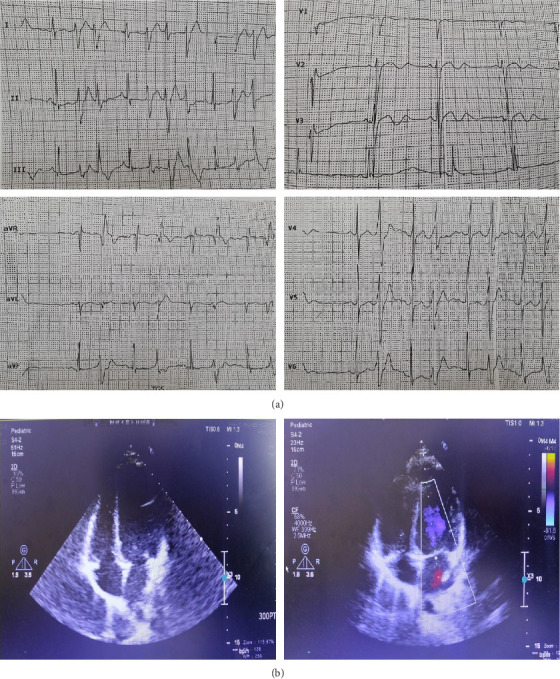
The clinical information of the patient is presented herein. (a) The electrocardiography panel shows QT interval prolongation, infrequent ventricular premature beats (single and coupled premature ventricular contractions), biphasic T waves, and prominent U waves in the anterior precordial leads. The heart rate, the PR interval, and the QRS duration are normal. (b) The echocardiography panel shows that all echocardiographic parameters, such as right and left ventricular systolic and diastolic function, are within the normal range. No significant dysfunction in the structural tissue or cardiac valves is detected.

The current study was conducted on a 3-generation Iranian family, one of whose members had an initial diagnosis of long QT syndrome. The father (50 y/o) and mother (42 y/o) were consanguineously married and had 4 daughters and 2 sons. The proband was a 12-year-old girl, the third offspring of the family. The patient was referred to our center after experiencing several cardiac arrhythmia events. Her past medical history indicated hypothyroidism, recurrent periods of muscle weakness, and impaired exercise tolerance. A thorough physical examination showed a broad forehead and nose; a thin upper lip; mild facial asymmetries; cataracts; a palatine cleft; malar, maxillary, and mandibular hypoplasia; dental anomalies; and scoliosis. Her lab data revealed hypothyroidism without blood serum electrolyte imbalances. Twelve-lead electrocardiography (ECG) demonstrated a prolonged corrected QT interval (Figure 1(a)), and 24-h Holter monitoring showed a daily average of 5049 premature ventricular complexes, 45.08% of them being episodes of bigeminy and polymorphic premature ventricular complexes, and 0.65% episodes of no sustained polymorphic ventricular tachycardia. The monitoring also revealed 6 sustained polymorphic ventricular tachycardia episodes, indicating torsade de pointes, and 601 supraventricular premature beats. Echocardiography illustrated no cardiac abnormalities in terms of valvular function or structural tissues (Figure 1(b)). The patient's drug history included metoprolol for long QT-interval control and vitamin supplement tablets.

The other family members underwent physical examinations, ECG, echocardiography, and genetic tests, which disclosed no abnormalities. Due to the gap in data concerning our patient's definitive diagnosis, we decided to analyze her genetic features by Whole-exome sequencing (WES). The protocol of the present investigation complies with the Declaration of Helsinki. Ethical approval was obtained from the Ethics Committees of Rajaie Cardiovascular Institute, Tehran, Iran (IR.RHC.REC.1402.003). Written informed consent was obtained from a parent and/or legal guardian for study participation.

### 2.2. WES and Bioinformatics Analysis

**Figure 2:**
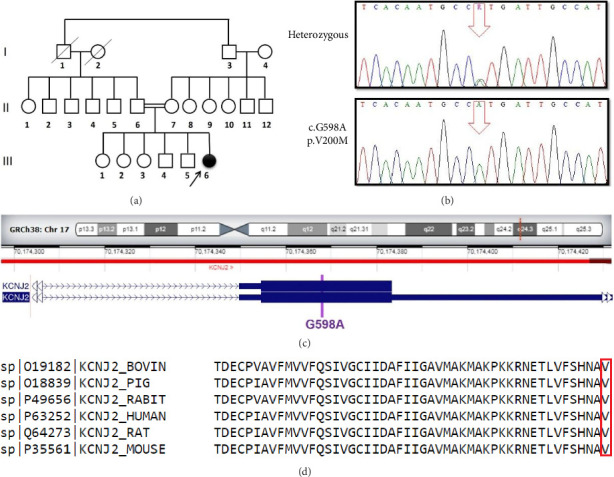
The image depicts the identification of a homozygous likely pathogenic *KCNJ2* missense variant (c.G598A: p.V200M). (a) The pedigree of the investigated family is presented herein. The filled symbols indicate the affected individuals. The arrow points to the affected proband (III-5). The double horizontal lines demonstrate consanguinity (II-6 and II-7). The slash points out the dead family members (I-1 and I-2). (b) The sequence chromatograms of the changed position of the *KCNJ2* gene show a homozygote state for the affected individual (III-5) and normal (III-1 and III-4) and heterozygous (III-2, III-3, and III-5) states for the healthy individuals. (c) The genomic structure of *KCNJ2* is illustrated herein, indicating the graphical representation of the linkage interval in the pericentromeric region of Chromosome 17, and the position of *KCNJ2*. Nucleotide variant (purple) of *KCNJ2*, indicating the variant identified in this study (c.G598A), is placed on the gene prediction region. (d) The image demonstrates the amino acid alignment of *KCNJ2* in humans and different species. The variant identified in this study is marked in red.

For an accurate diagnosis, peripheral blood samples were collected from the proband and all her available family members (Figure 2(a)). Genomic DNA extraction was performed using an in-house salting out method. The quality of each DNA sample was controlled with a NanoDrop 2000 spectrophotometer (Thermo Fisher Scientific), and 10 ng of the genomic DNA was used for WES on a HiSeq6000 sequencer (Illumina, San Diego, CA, USA). WES for the proband was performed at Macrogen (Amsterdam, Netherlands), and raw data were analyzed at Rajaie Cardiovascular Medical and Research Center, Tehran, Iran. The quality of the FASTQ file was checked using the FastQC software (V.0.12.0). The sequence reads were aligned with the human genome reference (the National Center for Biotechnology Information [NCBI], hg19) using the Burrows–Wheeler Aligner (V.0.6), and variant calling was performed using the SAMtools software (V.0.1.x). The variants were annotated using the ANNOVAR software, and finally, filtering and prioritization were carried out. For the bioinformatics analysis, the pathogenicity of the detected variant was predicted using the American College of Medical Genetics and Genomics (ACMG) [8], Polymorphism Phenotyping v2 (PolyPhen-2), Sorting Intolerant From Tolerant (SIFT), MutationTaster, and Combined Annotation–Dependent Depletion (CADD) tools. In addition, HOPE (https://www3.cmbi.umcn.nl/hope/input/) was utilized to collect more information about the mutant protein. The domains of the Kir2.1 protein were obtained from the Pfam database. The 3D structure of the protein was acquired using the Protein Data Bank and NCBI. The conservation of the variant region was checked using the ClustalW software (V.2.1).

### 2.3. Polymerase Chain Reaction (PCR) and Sanger Sequencing

Segregation analysis of the identified variant was performed using Sanger sequencing (Figure 2(b)). With the aid of the Gene Runner software and the Primer-BLAST primer design tool on the NCBI database, primer pairs were designed for PCR reactions. Whole exons, including the candidate variant, were amplified by PCR. The PCR reaction mixture contained 10 μL of Master Mix 1.5X (Applied Ampliqon, ID No. 5200350), 0.5 μL of 10 pmol of each primer, 1 μL of 200 ng DNA, and 8 μL of H_2_O. The PCR reactions were composed of initial denaturation at 95°C for 5 min, 35 cycles of denaturation at 95°C for 30 s, annealing at 63°C for 30 s, extension at 72°C for 30 s, and final extension at 72°C for 10 min. The length of the amplified segment was 472 base pairs. The PCR products were sequenced directly on an ABI3500 DNA sequencer (Thermo Fisher Scientific, Waltham, MA, USA).

## 3. Results

### 3.1. Clinical Analysis

The proband's physical examination revealed distinct facial and skeletal features, including short stature; a broad forehead and nose; mild facial asymmetries; a thin upper lip; a palatine cleft; malar, maxillary, and mandibular hypoplasia; dental anomalies; cataracts; hypertelorism; slight bilateral ptosis; low-set ears; scoliosis; periodic paralysis; and motor weakness in all the extremities. She also had an intellectual delay and palpitations without syncope. In contrast, the other family members were asymptomatic. Her cardiac echocardiography, with the exception of mild regurgitation, was normal insofar as she had a left ventricular ejection fraction of 55% with normal systolic and diastolic function. In addition, the 2D echocardiographic parameters of all 4 cardiac chambers were within the normal range. A review of the proband's 12-lead ECG demonstrated characteristic ECG features, such as QT-interval prolongation, biphasic T waves in the precordial leads, and a prominent U wave in V_1_–V_6_, which in infrequent parts led to ventricular premature beats of nonsustained ventricular tachyarrhythmia. The corrected QT interval was 470 ms. Ambulatory 24-h ECG tracings showed sinus rhythm with premature ventricular beats in a bigeminal pattern and in some episodes, runs of polymorphic and bidirectional ventricular tachycardia.

### 3.2. Molecular and Bioinformatics Analyses

**Figure 3:**
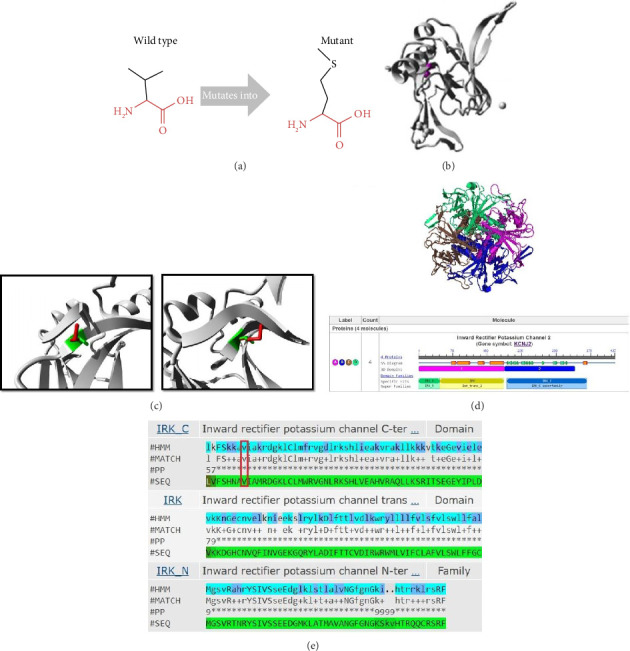
Schematic structures of the wild and mutant *KCNJ2* amino acids are presented herein. (a) Red: the backbone and black: the unique side chain. The residue of the mutant is larger than the residue of the wild type. The wild-type residue is well conserved. The mutated residue is also located near a highly conserved position. (b) The image presents an overview of the protein in the ribbon presentation. Gray: the protein and magenta: the side chain of the mutated residue shown as small balls. (c) The image is a close-up of the variant. Gray: the protein, green: the side chains of the wild-type residue, and red: the side chains of the mutant residue. (d) The 3D structure of the KCNJ2 protein and its molecular components is illustrated herein. Pink: Chain A, blue: Chain B, brown: Chain C, and green: Chain D. (e) The image presents the protein sequence and domain for Pfam matches. IRK_C: inward rectifier potassium channel C-terminal domain, IRK: inward rectifier potassium channel transmembrane domain, and IRK_N: inward rectifier potassium channel N-terminal. Based on the protein sequence, the identified variant is located in the C-terminal region of the Kir2.1 protein.

We identified a novel homozygous likely pathogenic missense variant (chr17, c.G598A, p.V200M; hg19; NM_017755.5) (Figure 2(c)) in the proband (Figure 2(a): III-6). This DNA change was predicted as disease-causing and protein-function altering by SIFT; likely pathogenic and probably damaging by ACMG and PolyPhen-2, respectively; and had a CADD Phred score of 24.0. The variant was confirmed in the patient as homozygous (Figure 2(b)). The parents were heterozygous in this position (Figure 2(a): II-6 and II-7), 2 of the siblings had normal sequences (Figure 2(a): III-1 and III-4), and the 3 others were heterozygous (Figure 2(a): III-2, III-3, and III-5). The wild-type residue was well conserved, and the mutant residue was not among the other residue types observed in other homologous proteins in this region. ClustalW predicted a widely conserved nucleotide position (Figure 2(d)). The HOPE results (Figures 3(a), 3(b), and 3(c)) showed that the wild-type and the mutant amino acids were different in size, and in that the mutant residue was larger, and the wild-type residue was covered in the core of the protein. A 3D structure was obtained using the Protein Data Bank (ID = 7ZDZ ID) (Figure 3(d)), which demonstrated that the wild-type residue was located in a β-strand, the preferred secondary structure. The mutated residue was present in the main domain, located in the highly conserved cytoplasmic C-terminal domain (Figure 3(e)) [9].

## 4. Discussion

The principal goal of all medical research is to reduce catastrophic events by enabling early diagnosis and identifying optimal therapeutic strategies. ATS is a rare autosomal dominant channelopathy characterized by a clinical triad of skeletal dimorphism, periodic muscular weakness, and cardiac manifestations due to prolonged QT intervals. While clinical presentation is essential for initial diagnosis, genetic confirmation remains the gold standard for definitive diagnosis. In our study, we observed classical features of ATS across three generations of an Iranian family, including long QT intervals, periodic paralysis, and skeletal anomalies, underscoring the relevance of molecular diagnostics in rare channelopathies.

Due to its low prevalence and variable expression, more than 90% of ATS cases lack the complete clinical triad, making diagnosis challenging [10]. Our proband represents a rare but informative case confirmed by molecular testing. WES revealed a novel homozygous variant in the KCNJ2 gene, a key gene associated with ATS. KCNJ2 encodes the inwardly rectifying potassium channel Kir2.1, which plays a central role in maintaining cardiac excitability [6, 11–13]. Mutations in this gene impair potassium conductance and disrupt the repolarization phase of the cardiac cycle, resulting in prolonged QT intervals and prominent U waves on ECG [14]. The mutant KCNJ2 variant in our patient also accounts for her muscle weakness and periodic paralysis, as potassium imbalance is known to exacerbate arrhythmias and muscle symptoms. Several studies have highlighted the importance of potassium supplementation in managing these features [3, 7]. However, no standardized therapy currently exists for ATS. Management strategies include potassium tablets, carbonic anhydrase inhibitors such as acetazolamide, and lifestyle modifications to avoid triggers of paralysis. These interventions aim to stabilize cardiac function and reduce neuromuscular symptoms, although the prognosis remains variable due to arrhythmic risks [6, 10, 11].

Most ATS-associated KCNJ2 variants produce nonfunctional channels and exert a dominant negative effect on the function of the native Kir2.1 protein [1, 15–17]. This channel causes long-term depolarization of the action potential, causing periodic paralysis and cardiac arrhythmias [18]. Furthermore, ATS-associated variants modify the sensitivity of Kir2.1 to phosphatidylinositol-4,5-bisphosphate (PIP2), a necessary activator for the channel. Consequently, about 50% of variants are in residues essential for the interplay between Kir2.1 and PIP2 [13, 19, 20].

In our patient, the *KCNJ2* c.G598A (p.V200M) variant was in the C-terminal intracellular domain of the Kir2.1 channel, a region essential for its interaction with PIP2, a phospholipid necessary for channel activation [9, 16, 21, 22]. Functional studies have shown that variants in this region may exert dominant negative effects and severely reduce channel activity. Interestingly, while the proband was homozygous for this variant, her heterozygous family members exhibited no cardiac symptoms, suggesting incomplete penetrance or modifier effects. To our knowledge, this is the first reported case of a causative KCNJ2 variant in an Iranian family with ATS, contributing to the growing body of literature on genotype–phenotype correlations in this rare disorder.

## 5. Conclusions

The variants causing the diverse presentations of ATS are in *KCNJ2* and *KCNJ5*, and the NGS sequencing role in correlating each phenotype with the exact gene is now more prominent than ever before.

## Data Availability

The datasets generated and/or analyzed during the current study are available in the ClinVar repository (https://www.ncbi.nlm.nih.gov/clinvar/variation/2502416/). The accession number of the variant in ClinVar is as follows: NM_000891.3 (KCNJ2): c.598G>A (p.Val200Met): VCV002502416.1.
